# Analysis of the Calcium Phosphate-Based Hybrid Layer Formed on a Ti-6Al-7Nb Alloy to Enhance the Ossseointegration Process

**DOI:** 10.3390/ma13235468

**Published:** 2020-11-30

**Authors:** Alicja Kazek-Kęsik, David Djurado, Stéphanie Pouget, Agata Blacha-Grzechnik, Izabela Kalemba-Rec, Wojciech Simka

**Affiliations:** 1 Faculty of Chemistry, Silesian University of Technology, B. Krzywoustego Street 6, 44-100 Gliwice, Poland; wojciech.simka@polsl.pl; 2 Université Grenoble Alpes, CEA, CNRS, IRIG/SyMMES/STEP, F-38000 Grenoble, France; davidju38@yahoo.fr; 3 Université Grenoble Alpes, CEA, IRIG/MEM/SGX, F-38000 Grenoble, France; stephanie.pouget@cea.fr; 4 Faculty of Chemistry, Silesian University of Technology, M. Strzody Street 9, 44-100 Gliwice, Poland; Agata.Blacha-Grzechnik@polsl.pl; 5 Faculty of Metals Engineering and Industrial Computer Science, AGH University of Science and Technology, A. Mickiewicza Avenue 30, 30-059 Krakow, Poland; kalemba@agh.edu.pl

**Keywords:** hybrid coatings, ceramic coatings, plasma electrolytic oxidation, titanium alloys, biomaterials, dental implants, bone tissue

## Abstract

This paper reports on hybrid, bioactive ceramic Ca-P-based coating formation on a Ti-6Al-7Nb alloy substrate to enhance the osseointegration process. The Ti alloy was anodized in a Ca_3_(PO_4_)_2_ suspension and then the additional layer was formed by the sol-gel technique to obtain a mixture of the calcium phosphate compounds. The oxide layer was porous and additional ceramic particles were formed after sol-gel treatment (scanning electron microscopy analysis coupled with energy-dispersive x-ray spectroscopy). The ceramic particles were formed on some parts of the oxide layer and did not completely fill the pores. The layer thickness of the anodized Ti alloy was comprised between 3.01 and 5.03 µm and increased to 7.52–12.30 µm after the formation of an additional layer. Post-treatment of the anodized Ti alloys caused a decrease in surface roughness, and the layer became strongly hydrophilic. Crystalline phase analysis (X-ray diffraction, XRD) showed that the hybrid layer was composed of TiO_2_ (anatase), Ca_3_(PO_4_)_2_, Ca_10_(PO_4_)_6_(OH)_2_ and a partially amorphous phase; thus, the layer was also analyzed by Raman spectroscopy. The hybrid layer showed worse adhesion to the substrate than the anodized layer only; however, the coating was not brittle, and the first delamination of the layer was determined at 1.84 ± 0.11 N during scratch-test measurement. The hybrid coating was favorable for collagen type I and lactoferrin adsorption, strongly influencing the proliferation of osteoblast-like MG-63 cells. The coatings were cytocompatible and may find applications in formation of the functional layers on long-term implants’ surface after.

## 1. Introduction

Medical metallic biomaterials are made of titanium (titanium Grade 4) or its alloy, Ti-6Al-4V. The surface of implants dedicated to bone is usually mechanically treated, machined, polished, sand-blasted or etched [1,2]. The formation of one nanostructured layer or porous layer on the implants influences interactions between the surface and bone tissue. The nanostructured surface of titanium alloys is formed by etching or alkali treatment of the implant at various temperatures and with various alkalis (e.g., NaOH and KOH) or acidic solutions (e.g., HCl, HF, H_3_PO_4_ and CH_3_COOH) [3,4]. Thin layers with open or closed pores (usually open) and irregular flakes may induce faster protein and water molecule adsorption and, thus, osteoblast cell adhesion. Thicker coatings with the included chemical compounds are formed using an electrochemical technique, such as anodization, including plasma electrolytic oxidation, electrophoretic deposition, electroreduction or electrodeposition. A good, adhesive layer on the substrate exhibits the oxides formed during plasma electrolytic oxidation (PEO) [5]. Additionally, during anodization, chemical compounds from electrolytes might be incorporated when forming the porous oxide layer [6]. The PEO process has limitations, such as the incorporation of ceramic particles into the layer or controlling the process to form an agglomeration of ceramic particles on the top layer. These particles at the top of the coatings play a key role in the interactions with proteins and osteoblast cells, which participate in the osseointegration process [7,8].

The formation of phosphate-based coatings on titanium alloys is well established. However, the final effect of the physico-chemical properties of the coatings and cytocompatibility of the surface vary. Hydroxyapatite (Ca_10_(PO_4_)_6_(OH)_2_), which is a material that influences osseointegration and osseoinduction, is often blended with other phosphates—e.g., Ca_3_(PO_4_)_2_ (TCP). Calcium phosphates are present in two polymorphic forms: the α-phase Ca_3_(PO_4_)_2_ is less soluble, and the β-phase Ca_3_(PO_4_)_2_ is more commonly used in biomaterials [9]. Biphasic ceramic materials comprising hydroxyapatite and tricalcium phosphate are formed as grains of various sizes, including nanoparticles or grains that might be deposited on the metal surface or mixed with polymer (synthetic) or biopolymer (natural) materials. The main goal of the biphasic HA/TCP is bioactivity, with favorable properties to form various shapes of the materials and the ability of resorption [10]. HA/TCP biphase material might be deposited using the plasma spraying technique on a Ti-6Al-4V surface [11] or on a Ti surface [12]; the material might also be blended with another chemical compound, where ions such as Zn^2+^, Ag^+^ and Cu^2+^ [13,14] also exhibit antimicrobial properties. Coating formation using the sol-gel technique allows the design of a surface with desirable chemical and phase compositions. This technique is used to form the hybrid layer and enrich the top, previously anodized titanium alloys. Titanium surface treatment may be provided by various techniques, such as magnetron sputtering and electrochemical deposition (formation of hydroxyapatite on Ti–Ta–Zr alloys) [15], pulsed laser deposition (F/Mg HA doped on Ti implant) [16] or via electron beam evaporations to form a hydroxyapatite layer on a β-phase Ti alloy [17]. Previously anodized surfaces of Ti alloys can also be modified by using the electrophoretic technique to enrich the top part of the layer in selected compounds, such as chitosan [18], graphene oxide (rGO)—bioglass [19] or apatite/biopolymer [20].

Another advantage of ceramic coatings is the easy adsorption of proteins, such as collagen type I or collagen type II, and lactoferrin, which stimulate the osseointegration process. Bone is a porous structure with various densities, depending on the type and the considered part of bone. The adsorption of biomolecular coatings comprising protein can stimulate peri-implant bone due to the formation of a microstructure favorable for the adsorption of water molecules and osteoblast cells [21]. The formation of extracellular matrix (ECM) supports cell proliferation and cell migration and the final results of bone-to-implant contact (BIC). It was reported that ECM comprises almost 90% of collagen type I, which induces osteoid and mineralization processes, and the mRNA expression of cellular proteins promotes bone healing formation [22]. Lactoferrin is a glycoprotein that is also used for surface treatment to promote the behavior of MG-63 osteoblast-like cells [23]. Lactoferrin promotes the osteogenic differentiation of SAOS-2 cells to enhance bone regeneration in vivo [24,25]. The final biological response of the cells depends on several factors, not only the chemical composition. Thus, the evaluation of new coatings and materials should be examined and characterized in depth. In the scientific literature, information about favorable surfaces for bone regeneration is available, usually in some ranges. A Ca/P ratio such as 1.67 is recognized as a ratio in hydroxyapatite that promotes the osseointegration process [26]. However, various materials comprising various concentrations of calcium phosphates or mixtures of apatite without the Ca/P ratio equaling 1.67 also promote the formation of new bone tissue [27,28].

The aim of this study is to form hybrid ceramic coatings to improve bioactive properties of the vanadium-free Ti-6Al-7Nb surface. The first step of surface treatment was the formation of a porous oxide layer with incorporated tricalcium phosphate (Ca_3_(PO_4_)_2_) on the Ti alloy using the plasma electrolytic oxidation process. Next, an additional coating was deposited via the sol-gel technique from a solution with precursors to form hydroxyapatite particles. The hybrid coatings were characterized in detail, particularly the surface morphology, cross-section and chemical and phase composition. To enhance adhesion and proliferation of the MG-63 osteoblast-like cells, the surfaces were immersed in protein solutions (collagen type I or lactoferrin).

## 2. Materials and Methods

### 2.1. Ti Alloy Surface Treatment

The Ti-6Al-7Nb alloy (BIMO Metals, Wrocław, Poland) was anodized in 0.1 M Ca(H_2_PO_2_)_2_ solution (Alfa Aesar, Kandel, Germany) with 150 g/dm^3^ Ca_3_(PO_4_)_2_ (Avantor, Gliwice, Poland) particles at 350 V. The current density applied during the anodization process was 150 mA/cm^2^. The sample possessed a cylindrical shape with a diameter of 9.5 mm and a height of 5 mm. The procedure of surface pretreatment and anodization was described in our previous paper [29]. Next, an additional sol-gel coating was formed on the previously anodized sample according to the following procedure: 1 M KH_2_PO_4_ with 0.67 M Ca(NO_3_)_2_ (Sigma Aldrich, Poznań, Poland) in aqueous solution was prepared, and then, NH_3_ aq. (Alfa Aesar, Kandel, Germany) was added to adjust the pH to 10. The solution was mixed for 48 h (aging), and then, the anodized sample was coated using the dip coating technique at a dipping and pulling speed of 0.2 cm/min. The sample was then dried at room temperature and heat treated for 30 min in a furnace at 660 °C with a controlled ramp rate (10 °C/min) in the air condition. The sample was treated in the furnace at 660 °C for 2 h, followed by cooling down to ambient temperature (in the furnace).

### 2.2. Surface Morphology and Wettability

The morphology of the modified sample was examined by scanning electron microscopy (SEM) (Hitachi, Tokyo, Japan; TM-3000; BSE mode; accelerating voltage, 15 kV). A cross-section of the samples was also prepared, and then, the samples were placed in epoxy resin and dried at room temperature. Next, the samples in the resin were ground using silicon carbide SiC paper up to 1500 gradation. A thin layer of gold (Cressington Sputter Coater 108 Auto; Cressington Scientific Instruments UK, Watford, UK) was deposited on the sample and the thicknesses of the layers were analyzed by SEM. The cross-section was analyzed using a scanning electron microscope (SEM) with energy-dispersive X-ray spectroscopy (EDX) (Phenom Pro-X) (applied voltage was 15 kV).

The surface wettability was analyzed by measuring the water contact angle (goniometer DataPhysics; OCA 15EC, Filderstadt, Germany). For this measurement, 0.2 µL of deionized water was dropped onto the investigated surfaces and the contact angle was measured. The results of the water contact angle measurements were expressed as the average and standard deviation of three independent samples of the same type.

### 2.3. XRD Analysis

Powder X-ray diffraction (XRD) was performed using a Panalytical X’Pert powder diffractometer (Malvern Panalytical Ltd., Malvern, UK) equipped with a copper anode (λKα1 = 1.5406 Å and λKα2 = 1.5444 Å) and an X’Celerator 1D detector. It was configured in Bragg-Brentano geometry, with a variable divergence slit on the primary beam path and a set of anti-scattering slits positioned before and after the sample. Axial divergence was limited by 0.02-rad Soller slits.

### 2.4. Raman Spectroscopy

Raman spectroscopy was applied for determining the chemical composition of the hybrid coatings formed on the titanium alloy surface. Raman spectra were collected with a laser excitation at 614 nm and 1200 grating lines using a ViaRenishaw Raman microscope (Renishaw, Gloucestershire, UK) equipped with a charge-coupled device (CCD) detector and a Renishaw Macro Sampling kit with an Olympus LMPlanFl 50× lens. The measurements were carried out in the range of 200–1600 cm^−1^. The Raman spectra were subjected to the procedures of smoothing and baseline subtraction using Renishaw’s Windows^®^-based Raman Environment (WiRE™, Berlin, Germany) software WiRE2.

### 2.5. Scratch Test

The adhesive strength between the coating (TAN-SG) and substrate (titanium alloy) was evaluated using a Micro Scratch Tester (MST, CSM Instruments, Waltham, MA, USA) CSM instrument applying a Rockwell ‘C’ diamond indenter (serial number: J-147; 100-µm radius) at 30 mN–20 N loads at the length of 5 mm and a 2.5-mm/min table speed. The results of the scratching test were expressed by determining the critical load and coating delamination according to a previously described procedure [30].

### 2.6. Cytocompatibility

The investigated samples without protein were sterilized by autoclaving at 134 °C for 20 min. The sterile samples were immersed in protein solution (lactoferrin or collagen type I) and placed under a UV lamp for 10 min. Evaluation of titanium alloy sample cytocompatibility was carried out using human osteoblast-like MG-63 cells (European Collection of Cell Cultures, Salisbury, UK), seeded at an initial density of 8000 cells per sample in 1 mL of EMEM (Eagle’s minimal essential medium) cell culture medium (American Type Culture Collection, Manassas, VA, USA) supplemented with 10% FBS, 1% penicillin/streptomycin and 0.1% amino acids and sodium pyruvate (PAA, Worms, Germany) at 37 °C under a humidified atmosphere with 5.0% CO_2_. The samples with cells were incubated for up to 7 days. After 24 h, 3 days and 7 days, 50 µl of Alamar Blue reagent was added in phosphate-buffered saline (PBS) solution to the culture medium, and the cells were incubated for 4 h. Cell metabolic activity was evaluated according to the reduction of the Alamar Blue reagent, which was measured fluorescently (FLUOstar Omega; BMG Labtech, Ortenberg, Germany) as described previously [31]. The results were expressed as the average and standard deviation (S.D.) of three independent samples. The Student’s t-test was used for statistical analysis of the differences between the studied groups. 

The samples were also immersed in two different phosphate-buffered saline (PBS) solutions, comprising collagen type I (rat tail; purity ≥ 90% by SDS-PAGE; BD Biosciences, Poland) or lactoferrin (human; purity ≥ 90% by SDS-PAGE; Sigma Aldrich, Poznań, Poland) at 0.200 mg/mL. Next, 1 mL of the solution containing protein was incubated at 37 °C and shaken at 80 rpm. The viability, attachment and distribution of the adhered cells were evaluated using live/dead staining and a mixture of 2 μL (1 mg/mL) of calcein AM (Sigma) and 2 μL (1 mg/mL) of propidium iodide (Sigma). The samples were washed with PBS and examined under a fluorescence microscope (Zeiss Axiovert 40; Carl Zeiss, Jena, Germany). On images, live cells were stained green while dead cells were stained red. 

## 3. Results

The surface of the Ti-6Al-7Nb sample (TAN) was anodized (TAN-A) in calcium hypophosphite solution with the addition of ceramic particles (Ca_3_(PO_4_)_2_). Next, the samples were coated with an additional ceramic layer using the dip coating method to form additional bioactive particles (TAN-SG sample). The sample labels and process of the surface treatment are collected in Table 1.

**Figure 1:**
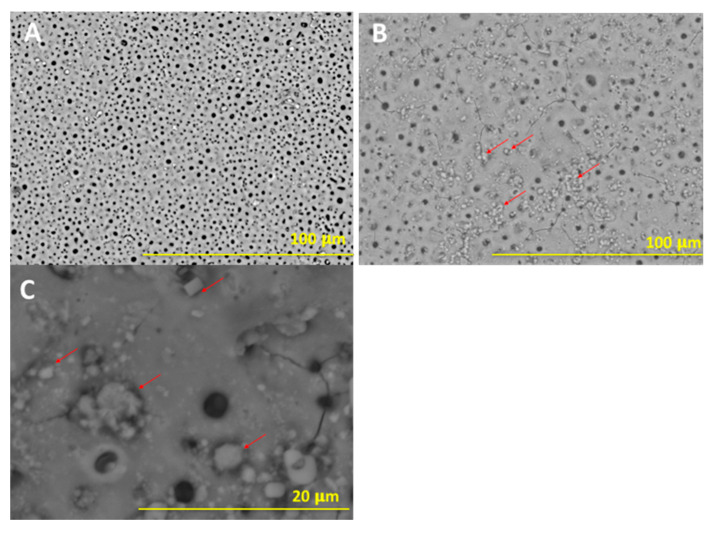
SEM images of the surface morphology of the (**A**) anodized sample, TAN-A, anodized sample coated with an additional ceramic layer, TAN-SG (**B**), and the TAN-SG sample (**C**) presented at higher magnification. The ceramic particles formed on the top of the layer are marked by red arrows.

Figure 1 presents the surface morphology of the TAN-A sample (A), where a typical porous microstructure is observed. 

**Figure 2:**
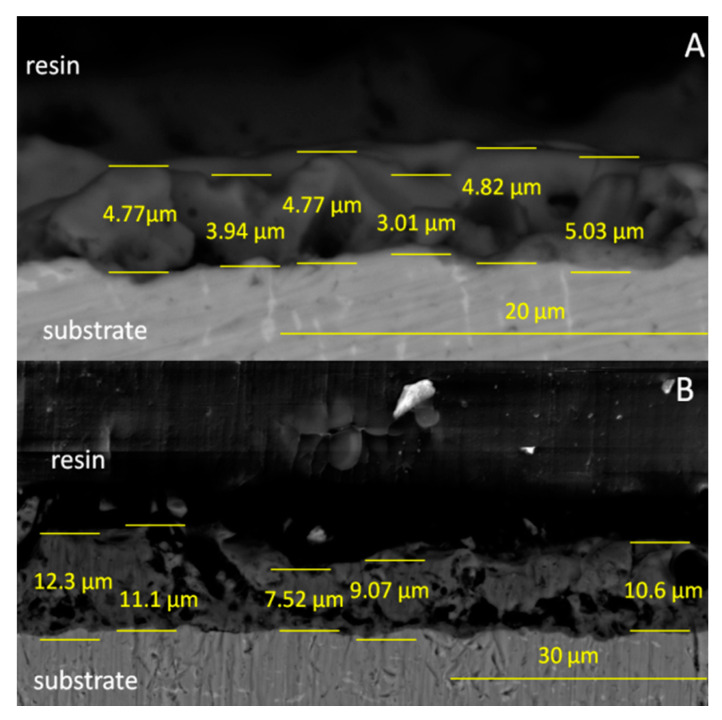
SEM image of cross-sections of (**A**) TAN-A and (**B**) TAN-SG samples.

The pore size and distribution depended on the parameters during the plasma electrolytic oxidation process, such as voltage, current density and the composition of the anodizing bath. The pores were distributed on the top of the layer but also through the layer as a result of the plasma electrolytic oxidation process. On the surface, single ceramic particles were observed due to the incorporation of Ca_3_(PO_4_)_2_ particles from the bath. It is possible that some ceramic particles were synthesized and built in the coating from the calcium hypophosphite anodizing bath (as discussed in the XRD results). Figure 1B,C show the morphology of the anodized sample post-treatment via the sol-gel technique (TAN-SG sample). Agglomerations of the ceramic particles formed due to the post-treatment technique are indicated with red arrows. The ceramic particles filled some of the pores, partly covering the oxide layer. Agglomeration of the ceramic particles was observed on the SEM image at a higher magnification (Figure 1C). The particles presented various distributions and did not cover the characteristic porous structure of the oxide layer. The thickness of the hybrid coatings formed on the Ti-6Al-7Nb surface was analyzed using SEM, and images of the cross-section of this sample are presented in Figure 2.

**Figure 3:**
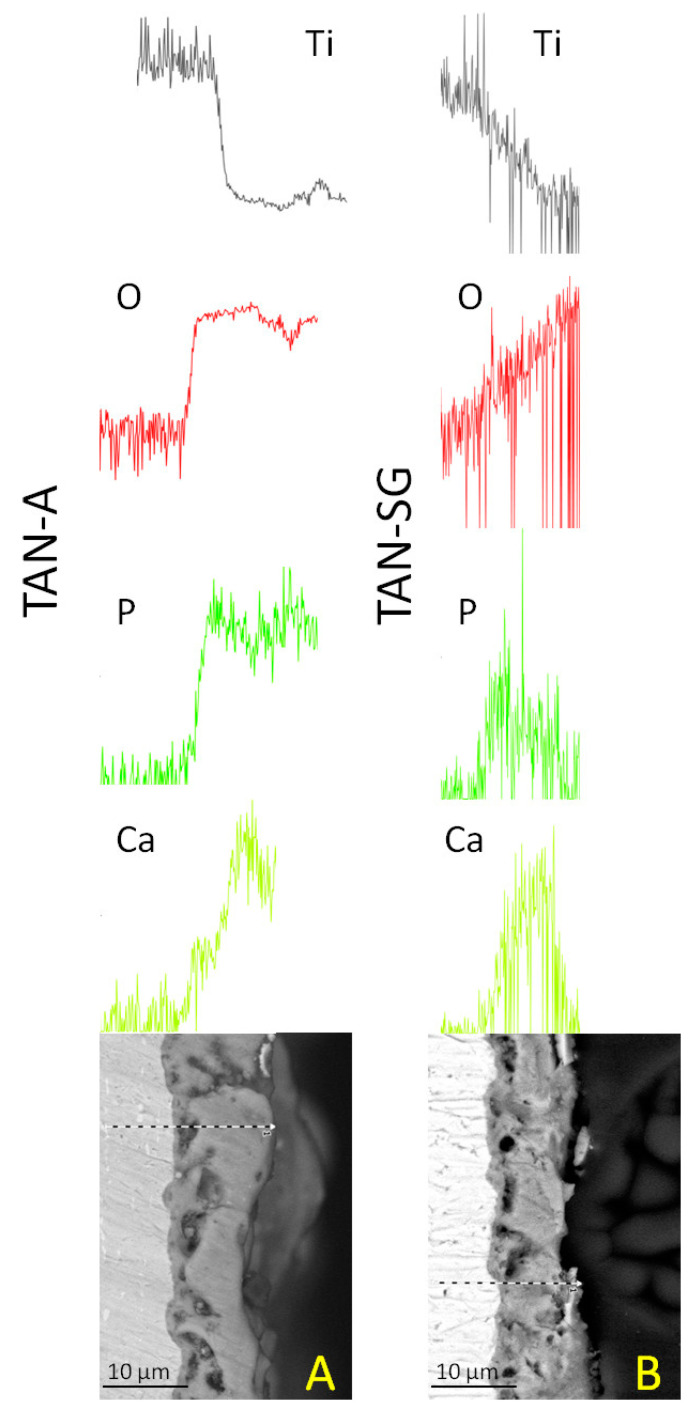
SEM images and energy-dispersive X-ray spectroscopy (EDX) analysis of (**A**) TAN-A and (**B**) TAN-SG samples**.**

The thickness of the oxide layer (TAN-A sample) was found in the range of 3.01–5.03 µm. The irregular shape of the oxide with open and closed pores is typical for the anodized Ti-6Al-7Nb alloy. The thickness of the hybrid ceramic layer was between 7.52 and 12.30 µm. The formation of the additional ceramic particles indicated that the total coating thickness was significantly increased. The morphology of the coatings also changed, and it is clearly visible that the coating is less porous and the sol-gel penetrated the pores of the oxide layer during the dip coating process. Figure 3 presents the cross-section and EDX analysis of the coatings.

The EDX lines and element distributions of calcium, phosphorous and oxygen confirmed the formation of ceramic coatings on the TAN substrate. For the TAN-A sample, the calcium and phosphorous are distributed more uniformly than for the, TAN-SG sample. For the sample after the sol-gel process, Ca and P compounds are more concentrated in the middle of the coatings and in some parts on the top of the coating. Detection of Ti and O elements indicated that TiO_2_ was formed on both samples. EDX lines of these elements show that in the layers, the content of Ti decreased whereas content of oxygen increased. 

**Figure 4:**
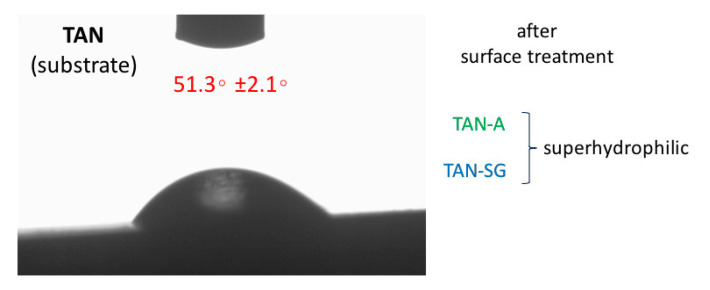
Water contact angle of the investigated samples. After surface treatment, the porous layers were superhydrophilic and the contact angle could not be determined.

Figure 4 presents the results of water contact angle measurements of the drop placed on top of the investigated samples. The wettability of the substrate (Ti-6Al-7Nb) sample was 51.3 ± 2.1°. Surface treatment of the TAN sample showed that the wettability was changed and the surface was superhydrophilic. The contact angle could not be determined due to fast adsorption of the water drop into the porous structure of the layers (for both samples, TAN-A and TAN-SG).

**Figure 5:**
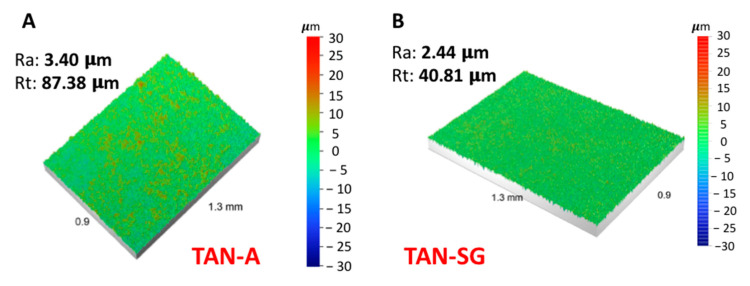
Optical images of the surface roughness measurements of (**A**) the TAN-A sample and (**B**) the TAN-SG sample.

The change in surface wettability also depends on the surface roughness. Figure 5 presents the results of the surface roughness analysis using a noncontact optical profilometer.

The anodization process of the titanium alloy (TAN sample) at 350 V indicated that the surface roughness Ra (average arithmetical parameter) increased from 0.30 to 3.40 µm. Next, the formation of the additional ceramic layer decreased the Ra parameter to 2.44 µm. The profile of the measured coating also decreased from 87.38 to 40.81 µm. Sol-gel treatment of the previously anodized Ti alloy indicated that some pores were filled by ceramic particles and the final measurement of the average surface roughness (Ra) decreased. 

**Figure 6:**
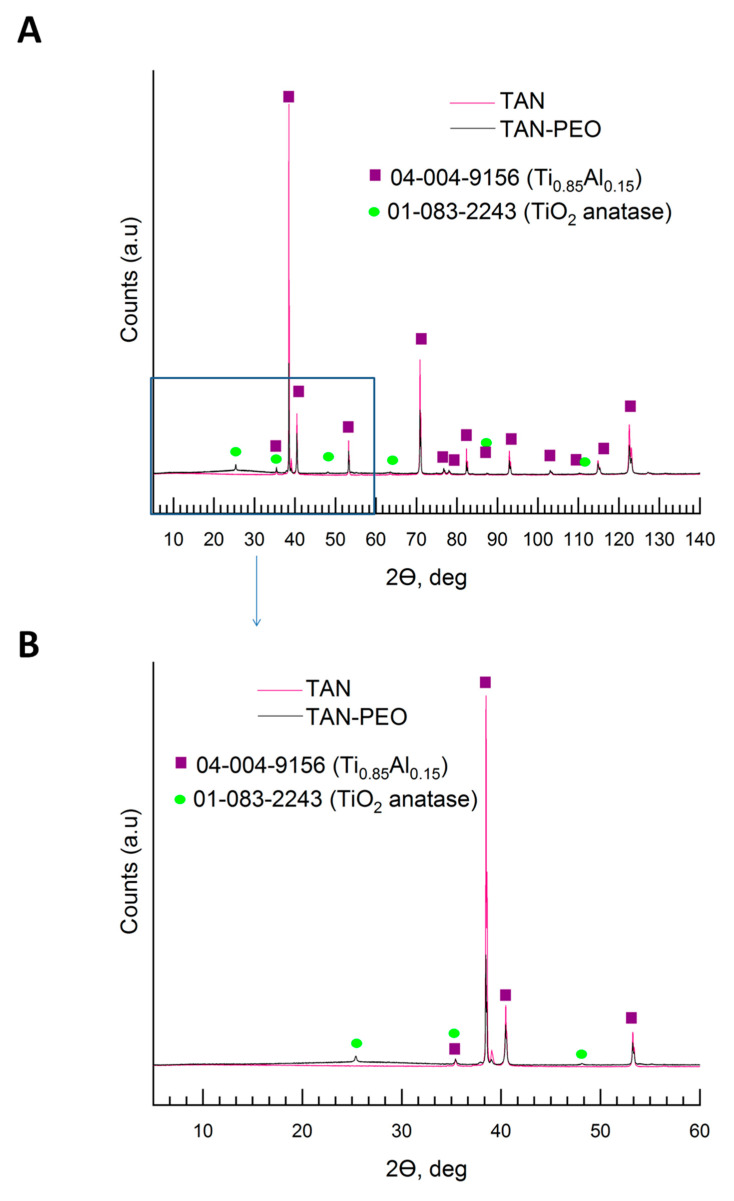
X-ray diffraction (XRD) pattern (**A**) of the TAN sample and the anodized TAN sample in solution without Ca_3_(PO_4_)_2_ particles at 350 V, and the same pattern (**B**) in range of 2θ 5–60°.

**Figure 7:**
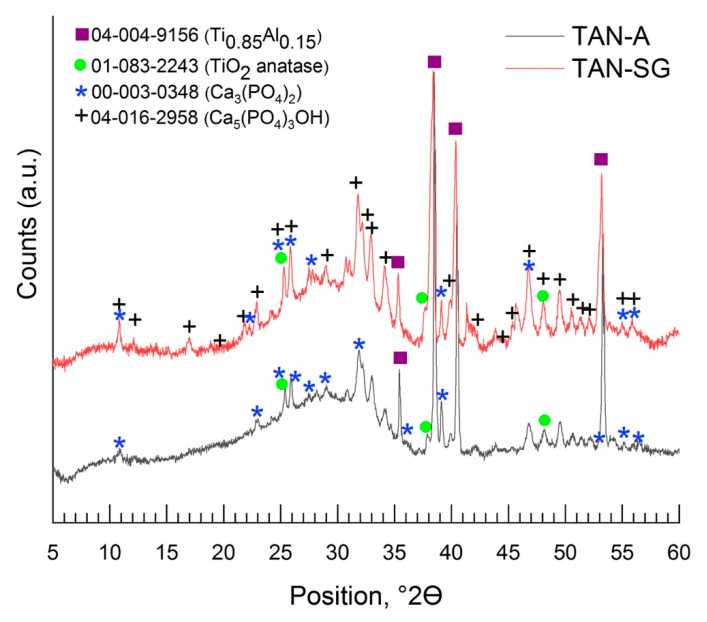
XRD pattern of the anodized TAN sample in solution with Ca_3_(PO_4_)_2_ particles at 350 V (TAN-A sample) and TAN-SG sample.

Figure 6 and Figure 7 show the XRD patterns of the coatings formed on the Ti-6Al-7Nb alloy. The phase composition of the layers formed on the Ti alloy was characterized step by step; it means that besides the substrate measurements, the coating formed in Ca(H_2_PO_2_)_2_ without ceramic particles was evaluated, then the coating formed in solution with Ca_3_(PO_4_)_2_ (TAN-A) and then the last one, the hybrid ceramic coating, which was formed due to anodization in solution with Ca_3_(PO_4_)_2_ particles and then coated with an addition layer via the sol-gel method (TAN-SG). For the Ti-6Al-7Nb titanium alloy, two phases were found: the phase of titanium and that of aluminum alloy (PDF #04-004-9156), where the main peak positions on the XRD pattern were detected at 35.40°, 38.47°,40.45°, 53.94°, 63.34°, 70.87°, 74.88°, 76.74°, 78.03°, 82.60°, 92.91° and 103.05° of 2θ. Anodization of the Ti-6Al-7Nb alloy in 0.1 M Ca(H_2_PO_2_)_2_ solution caused the formation of the titanium oxide—TiO_2_ anatase (PDF #01-083-2243) [32] (Figure 6). The main peaks for the titanium oxide were registered at 25.36°, 38.47°, 39.01°, 53.24°, 55.12°, 70.87° and 82.34° of 2θ.

No signals for the crystalline Ca-based phase were identified. The calcium phosphate was determined where the samples were anodized in the 0.1 M Ca(H_2_PO_2_)_2_ solution with ceramic particles of calcium phosphate (Ca_3_(PO_4_)_2_ (PDF #00-003-0348), and the XRD pattern is presented in Figure 7 [33].

The signals registered between 20° and 30° of 2θ indicated that the ceramic particles were incorporated. The peaks which correspond with the Ca_3_(PO_4_)_2_ phase was detected mainly at 25.96°, 28.24°, 31.89° and 37.21° of 2θ. However, an amorphous phase was also observed. During the plasma electrolytic oxidation process, XRD showed that the ceramic particles were melted, as revealed by the amorphous scattering background. The XRD pattern of the hybrid coating TAN-SG sample (anodized sample coated by the sol-gel technique) is shown in Figure 7. The signals from Ca-based compounds increased and new signals were determined. The new signals corresponded to calcium phosphate hydroxide (hydroxyapatite) (PDF #04-016-2958) [34] and calcium phosphate Ca_3_(PO_4_)_2_ (PDF #00-003-0348). For the hydroxyapatite, the main peaks are visible on the XRD pattern at 10.82°, 18.80°, 21.82°, 22.88°, 25.28°, 25.83°, 28.80°, 31.76°, 32.16°, 34.16°, 35.31°, 37.74°, 43.84°, 46.74°, 48.04°, 49.46°, 50.51° and 53.18° of 2θ. The signals of the anatase phase decreased due to formation of the additional ceramic layer, and the X-ray was mainly scattered by the layer formed by the sol-gel technique.

**Figure 8:**
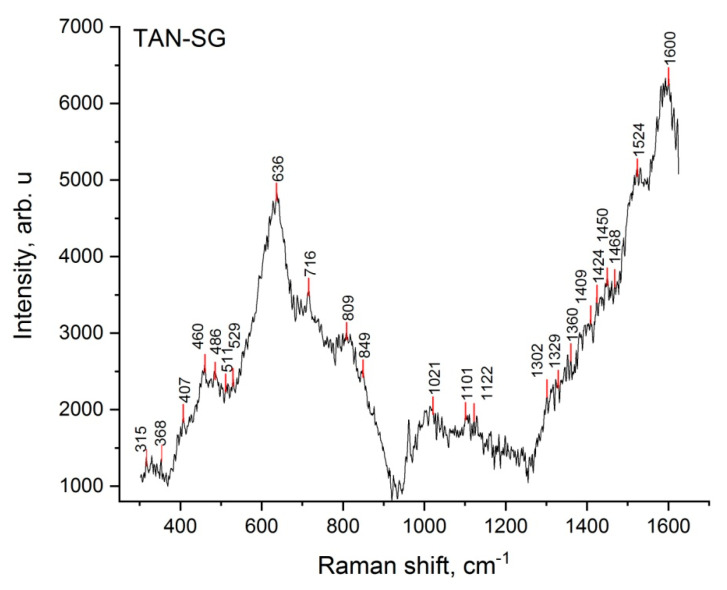
Raman spectrum of the TAN-SG sample.

To better characterize the TAN-SG sample, Raman spectroscopy was applied to determine the chemical composition of the amorphous and crystal parts of the layers. Figure 8 presents the Raman spectrum of the TAN-SG layer. 

The most characteristic vibrational spectra were registered for PO_4_^3−^ tetrahedra, which correspond to both ß-tricalcium phosphate (Ca_3_(PO_4_)_2_) and hydroxyapatite (Ca_10_(PO_4_)_6_(OH)_2_) chemical compounds. The tetrahedron on phosphates in aqueous solutions corresponded to vibrations such as *v*_1_ = 938 cm^−1^, *v*_2_ = 420 cm^−1^ and *v*_3_ = 1017 and 567 cm^−1^ [35]. In our study, the vibrations were detected between 403 and 412 cm^−1^, with the maximum at 407 cm^−1^, and then small peaks at 561 and 938 cm^−1^. PO_4_^3−^ exhibits vibrations that span frequency ranges of 400–490, 570–625 and 1020–1095 cm^−1^. The clear visible signal at 962 cm^−1^ corresponds to the nondegenerate *v*_1_ mode of PO_4_^3−^, which corresponds only to the hydroxyapatite structure. The broad signals and small signals registered between 368 and 530 cm^−1^ correspond to the *v*_2_ modes for ß-tricalcium phosphate.

**Figure 9:**
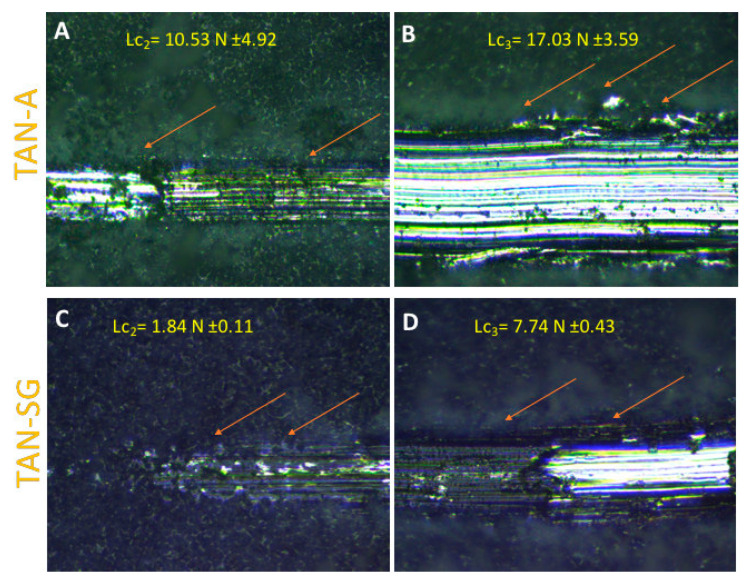
Optical images of the TAN-A (**A**,**B**) and TAN-SG (**C**,**D**) samples after the scratch test.

Figure 9 shows the evaluation of the ceramic coating adhesion to the substrate. The crack and layer during the first stage of the scratching of the coatings were not observed for both samples. According to the EN 1071-3:2005 standard [36], Lc_1_ is labeled when breakdown during the first stage of the intender penetrate is observed. Lc_2_ (plastic deformation of the layer) was determined for both layers (Figure 9A,C), occurring for the TAN-A sample when the friction force was 10.53 N ± 4.92 and for TAN-SG when the friction force was 1.84 N ± 4.0.11. For this layer, changes in the corner of the scratched part of the layer were observed (marked by red arrows). Lc_3_ is determined where the substrate is observed and the coatings are delaminated. For the TAN-A sample, the Lc_2_ and Lc_3_ were close and it was difficult to identify the raw substrate; thus, the final value for Lc_3_ was determined at 17.03 N ± 3.59. For the TAN-SG sample, Lc_3_ was observed when the friction force was 7.74 N ± 0.43.

**Figure 10:**
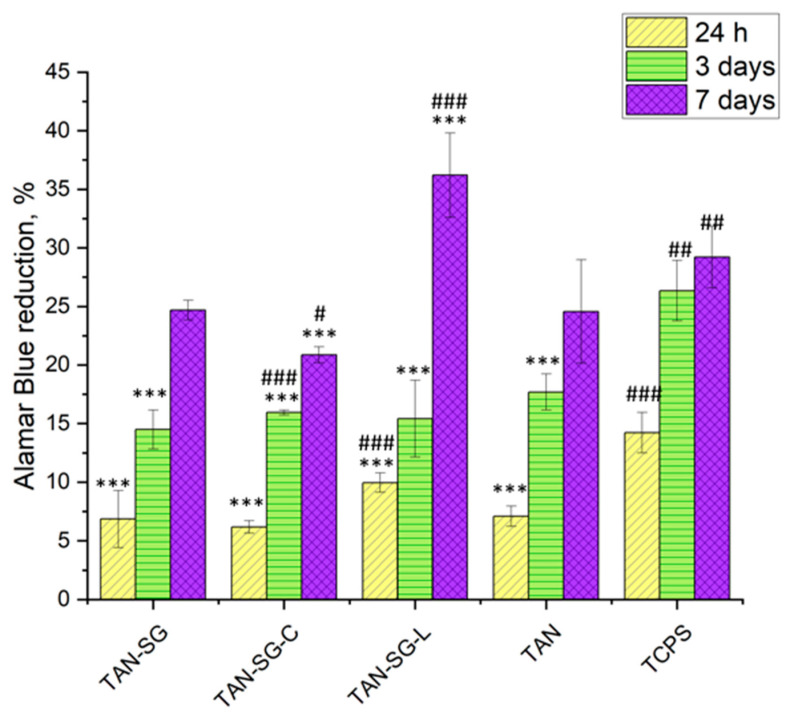
MG-63 cell viability after 24 h, 3 days and 7 days of culture in the investigated samples. The data are expressed as the mean ± S.E.M. of three similar experiments performed in triplicate. The (*) symbol indicate statistical significance from the control tissue culture polystyrene (TCPS) group, and from the non-modified surface TAN group, this was marked as (#); where *, # *p* < 0.05,**, ## *p* <0.01,***, ### *p* < 0.001.

Because the TAN-SG sample is to be used in a final application as a bone implant, biological characterization was provided for this surface. The TAN-SG sample was immersed in PBS solution containing 0.200 mg/mL of two different proteins—collagen and lactoferrin. The possibility of the adsorption of the protein and biological response was evaluated using MG-63 cells, particularly because the cell response was different after 3 and 7 days of the MG-63 cell culture. Figure 10 presents the viability of MG-63 cells cultured up to 7 days with the TAN-SG sample and the sample coated with collagen (TAN-SG-C) and protein (TAN-SG-L). The viability of the cells was also determined for the unmodified titanium alloy sample (TAN) and reference tissue culture polystyrene (TCPS). 

**Figure 11:**
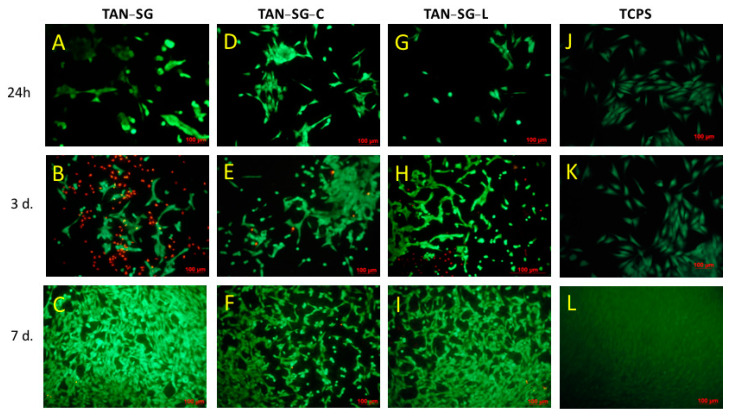
Optical images of MG-63 cells cultured up to 7 days with the TAN-PEO-SG (**A**–**C**) sample and the TAN-PEO-SG sample after immersion in collagen type I (**D**–**F**) or lactoferrin (**G**–**I**) solution. Images of the reference tissue culture polystyrene (TCPS) sample are marked as (**J**–**L**) after 24 h, 3 days and 7 days, respectively.

After 24 h of MG-63 cell culture, the highest number of cells was observed in the TAN-SG-L sample (besides reference TCPS). Surface modification, such as formation of a hybrid ceramic layer and immersion in proteins, influenced the differences in the number of cells compared with the unmodified TAN sample. After 3 days of culture, the cell number was similar for all the investigated samples. After 7 days, the highest cytocompatibility of the materials with MG-63 osteoblast-like cells was observed for the TAN-SG-L sample. Differences in the osteoblast-like MG-63 cells were observed after live/dead staining, and the optical images are presented in Figure 11. The live cells are visible in green and dead cells are visible in red. 

Optical images after osteoblast-like MG-63 cell staining showed effects of the protein on the cell morphology and cell viability. After 24 h on TAN-SG, some cell agglomeration was visible. When the TAN-SG sample was immersed in collagen type I (TAN-SG-C) and lactoferrin (TAN-SG-L) solution, the MG-63 osteoblast-like cells were better adhered. Few dead cells were observed. After 3 days of culture, many dead cells (red cells) were observed in the TAN-SG sample, whereas only single dead cells were visible on the surfaces after protein immersion. On all the surfaces, osteoblast-like MG-63 cells well adhered and their cell number increased with culture time. The highest number of cells was observed on the surfaces after 7 days of culture. 

## 4. Discussion

Anodization is a well-known method to modify valve metal surfaces. This phenomenon was mentioned in many studies, where the mechanism of the plasma electrolytic oxidation process is presented in detail [37,38]. Titanium alloys without vanadium, such as Ti-6Al-7Nb, not only enhance biocompatibility but also exhibit better (more favorable for bone tissue) mechanical properties compared to Ti Grade 4. The surface of long-term titanium implants for bone is modified to enrich the surface material with bioactivity. In our case, we focused on post-treatment of the previously anodized Ti-6Al-7Nb alloy to obtain a hybrid, bioactive ceramic layer. The SEM images show that on the porous layer, additional ceramic particles were formed after sol-gel treatment. Agglomeration of the ceramic particles occurred mainly in pores and on the top of the surface. The post-treatment analysis showed that the total layer thickness on the TAN-SG surface significantly increased. The EDX line analysis indicated that the sol penetrated the pores of the oxide layer and formed particles inside the oxide layer and on the top as well. For both samples, TAN-A and TAN-SG, the EDX line of oxygen and titanium indicated formation of the TiO_2_. A similar effect was observed for the alloying elements (Al, Nb) which are also anodized during the PEO process, but their content on the oxides in the total layer is low. Bioactivity is recognized as a process to enhance the formation of apatite comprising calcium phosphates with various ratios of Ca and P ions. It remains debatable whether hydroxyapatite presents higher bioactivity or tricalcium phosphate or a mixture of these materials and formation of biphasic ceramic material. Thus, post-treatment of the anodized surface is often performed to add specific functions of the materials—e.g., bioactivity or antibacterial properties [39,40,41].

Surface treatment of the T-6Al-7Nb alloy caused the surface roughness to increase, which is favorable for the implant to join bone tissue [42,43]. The results of the reference sample (TAN) surface roughness measurements were presented in our previous paper [29], where Ra was 0.30 µm and Rt was 0.15 µm. High surface roughness may lead to markedly decreased cytocompatibility of the material. Post-treatment of the anodized Ti alloys reduced their surface roughness from 3.40 to 2.44 µm. Hydrophilicity is a parameter that also positively affects the adsorption of proteins and then osteoblast cells and the osseointegration process [44]. For both surfaces, the wettability was highly hydrophilic and the contact angle could not be determined, but the surface of the TAN-SG sample was favorable for protein adsorption.

Based on previous studies, we hypothesized that calcium-based compounds (various, not only hydroxyapatite) are favorable for the osseointegration process. Calcium phosphate might be formed in crystalline or amorphous phase on top of the oxide layer; thus, Raman spectroscopy and the XRD technique were used for analyzing the coatings in detail. The broad shapes of the peaks observed in the Raman spectrum might be due to the high porosity of the coatings and formation of many OH^−^ groups. The signals at 638, 823 and between 315 and 330 cm^−1^ correspond to TiO_2_ anatase due to the anodization of the Ti alloy and were discussed in detail in our previous paper [45]. To understand the mechanism of formation the ceramic layer and results of cytocompatibility, the phase composition of the layers was analyzed in detail, step by step. First, the substrate was measured, where the phase of Ti._085_Al_0.15_ was found, with peaks close to that of the Ti phase. However, the maximum peaks of the recorded XRD patterns much better correspond to the phase with titanium and a small content of aluminum, such as the chemical composition of our Ti-6Al-7Nb alloys. The anodization process, only in Ca(H_2_PO_2_)_2_ solution, caused the formation of the oxide layer; however, some ions of calcium and phosphorous compounds were incorporated into the layer. The main part of the oxide layer comprises TiO_2_; in our case, the peaks correspond to the anatase phase. It is possible to obtain the layer with TiO_2_ in another polymorphic form, such as brookite or rutile, which was reported previously [46]. The differences in the polymorphic forms and the forms in coatings on the Ti alloy by the PEO process have been previously described [47]. The shape of the XRD pattern indicates that some amorphous parts were formed in the layer. No peaks from the crystalline calcium phosphate compounds were found. The peaks that corresponded to tricalcium phosphate (Ca_3_(PO_4_)_2_) were reordered when the Ti alloy was anodized in the solution (suspensions) with the ceramic particles of Ca_3_(PO_4_)_2_. In our case, some of the ceramic particles likely melted and were recorded by XRD as an amorphous-crystalline phase. Figure 7 shows that in our case, the amorphous parts on the XRD pattern increased. Some parts of ceramic particles were likely melted but existed inside the crystal and could be detected by XRD. Another explanation is the possibility to form calcium phosphates from the anodizing bath due to spark discharges. The temperature of the spark discharges could be different than those that occur during anodization in solution without ceramic particles. The formation of the additional ceramic layer by sol-gel revealed the hydroxyapatite and tricalcium phosphate phases (Figure 7). The peaks corresponding to the Ca_3_(PO_4_)_2_ phase was slightly shifted and less visible than those observed for the TAN-A sample. However, the peaks corresponding to the hydroxyapatite particles were well-defined at 2θ, indicating that this phase was formed during the post-treatment process (formation of the layer by the sol-gel technique). The chemical composition of the TAN-SG sample was also analyzed by Raman spectroscopy, which confirmed that the mixture of Ca_3_(PO_4_)_2_ and Ca_10_(PO_4_)_6_(OH_2_) was formed during the formation of the hybrid, ceramic coatings and formation of the calcium phosphate compounds from the anodizing bath. Formation of the compounds may be explained using the following reactions:

Ca(H_2_PO_2_)_2_ + H_2_O → Ca^2+^ + HPO_4_^2−^ + 4 H^+^ + OH^−^ (1)

Ca_2_P_2_O_7_ + H_2_O → 2 Ca^2+^ + 2 HPO_4_^2−^ (2)

3 Ca^2+^ + 2 PO_4_^3−^ → Ca_3_(PO_4_)_2_ (3)

10 Ca^2+^ + 6 PO_4_^3−^ + 2 H_2_O → Ca_10_(PO_4_)_6_(OH)_2_ + 2 H^+^ (4)

After the anodization process, Ca^2+^, HPO_4_^2−^ and OH^−^ probably exist in the layer. Thus, the formation of the additional ceramic layer cannot only explain the formation of the hydroxyapatite layer. The mechanism might be explained by reactions reported in a study [48].

Ca_10−x_(HPO_4_)_x_(PO_4_)_6−x_(OH)_2−x_(H_2_O)·*n*H_2_O → Ca_10−x_(P_2_O_7_)_x_(PO_4_)_6−2x_(OH)_2_(H_2_O)_x_ + *n*H_2_O (5)

Ca_10−x_(P_2_O_7_)_x_(PO_4_)_6−2x_(OH)_2_(H_2_O)_x_ + *n*H_2_O → *(*1 − *x)*Ca_10_(PO_4_)_6_(OH)_2_ + 3*x*Ca_3_(PO_4_)_2_ + 2*x*H_2_O (6)

In addition to the bioactivity of the surface, the mechanical properties of long-term implants play a crucial role in medicine. One method to evaluate the adhesion of the coating to the substrate is the scratch-test method. Oxide layers formed on a Ti alloy by the anodization process usually present good adhesion. This type of ceramic coating and the layer formed by the PEO process may be brittle. It is not obvious that all of the layers will be favorable for implant manufacturing due to their delamination during the implantation procedure. Takemoto et al. [49] reported that implanted implants with formed ceramic coatings (formed by the plasma-spray method) with a porosity of 40% showed a larger index of bone–implant contact with the bone tissue of rabbits than the non-treated surface of Ti. The differences in the affinity index were significant, and the final effect suggests that the layer was biocompatible and promising for clinical application. In our case, the layers were also porous. In our case, for all of the coatings, no characteristic signal for their brittleness was recorded (no acoustic emission signal). However, significant differences in their values of Lc_2_ and Lc_3_ were recorded. For the only-anodized surface (TAN-A) sample, the Lc_2_ and Lc_3_ values were higher, which indicates that the adhesion of the layer was much better than for the hybrid layer (TAN-SG). However, measurement of the porous coatings with higher surface roughness was less repeatable, and the highest standard deviation was calculated for the results of measurement of the TAN-A sample. Formation of the additional ceramic layer by the sol-gel process caused the thickness of the total layer to increase. However, the hybrid coating was not brittle and no cracks were observed during the scratch-testing. Surface roughness for this layer was lower, and repetition of the measurements showed better reproducibility. Beside the lower values of Lc_2_ and Lc_3_ for the TAN-SG sample, the results indicate that the layer was still well adhered. It is difficult to compare our results with others published in the literature due to differences in parameters applied during the scratching, applying different intenders and the thickness of the coatings, which play a key role in the coatings analysis [50]. The decrease in the adhesion of the TAN-SG sample could be caused by the heat treatment process, during which the porosity of the layer could change and moisture or other gases present inside the oxide layer might also evolve.

Adsorption of proteins influences the adhesion and proliferation of osteoblast-like cells. More than 28 types of collagen were recognized. For bone tissue treatment, one of them, collagen type I, is widely used as a commercial product, used to fill the bone hole as they are injected around the bones. Collagen types III, V and XXIV have been classified as fibril-forming, which contributes, with collagen type I, for example, in embryonic tissues and in cornea or plays a role in selective expression in developing cornea and bone [51]. Collagen type I exists mainly in the organic part of bone matrix. It participates in the remodeling process of bone tissue and it is used to modify the surface of ceramic materials, such as scaffolds. Collagen type I enhances osteoconductive properties and increases the surface area for osteoblast cell adhesion [52]. Different mechanisms of cell interaction with lactoferrin (LF) were recognized as well. It was found that LF promotes in vitro and in vivo osteoblast proliferation and survival and inhibits osteoclastogenesis as well [53]. It was reported that LF induces ERK (erk-kinase) phosphorylation in osteoblast cells. 

The main purpose of surface treatment with ceramic materials is to enhance the bioactivity of the surface. Immersion of the samples in the solution with the proteins (collagen or lactoferrin) influenced the osteoblast-like MG-63 cell adhesion and proliferation (see Figure 10 and Figure 11). It was clearly seen that after immersion of the TAN-SG sample in the lactoferrin solution, the cells were faster and much better adhered, so their proliferation could be observed. These results were obtained after 3 and 7 days of cell culture, meaning that the response is from the surface of the sample, not from the solution and culture medium, which could be slightly modified during the first 24 h of the cell culture (the proteins could diffuse from the surface to the culture medium). The fluorescence image indicates that on the TAN-SG sample surface, a number of dead cells (red cells) are observed after 3 days of the culture. However, the number of live cells is slightly lower (on the fluorescence images) than the number of cells observed on the TAN-SG sample after immersion in the solution of collagen type I or in the solution of lactoferrin. After 7 days of the culture, for all the surfaces, the cells were well adhered and only single dead cells were observed, which are usually detected during such in vitro experiments. Thus, we have concluded that lactoferrin supports the proliferation of osteoblast-like MG-63 cells. The ability of this protein to adsorb on ceramic coatings is a factor which potentially might be used to form the materials with LF to induce synthesis of growth factors and cytokines by osteoblasts. It was reported that hLF (human lactoferrin) exhibits anti-apoptotic properties towards MC3T3 pre-osteoblast cells [54]. The sample immersed in collagen type I solution (TAN-SG-C) did not present much better cytocompatibility with the cells; however, the surface was not cytotoxic. The TAN-SG sample without protein was also cytocompatible with osteoblast-like MG-63 cells up to 7 days of their culture. The surfaces were completely covered with the cells and only single dead cells occurred, likely resulting from the in vitro conditions and limited space for cell proliferation. Significant differences were observed between the surfaces up to 3 days of culture, in which immersion of the TAN-SG sample in lactoferrin solution positively influenced cell behavior, such as adhesion and proliferation. Application of osteoblast cell growth factors on the bone implants is one of the ways to promote osseointegration and to promote adhesion of osteoblast cells on the implant surface faster than, for example avoid septic inflammation which may occur after the surgery. 

## 5. Conclusions

The results indicate that an additional ceramic layer containing hydroxyapatite can be grown on anodized Ti-6Al-7Nb alloy as a substrate. We have found that both the layer formed by anodizing and the ceramic layer formed by the sol-gel technique are composed of amorphous and crystalline phases. The main phase composition of the anodized layer is TiO_2_ (anatase), and incorporated particles from suspension Ca_3_(PO_4_)_2_ were detected by XRD technique. Some parts of the ceramic particles could be also formed during the spark discharges occurring during the PEO treatment. Formation of additional ceramic layer by sol-gel decreased the surface roughness of the anodized layer. The wettability became strongly hydrophilic, which was favorable for adsorption of collagen type I (rat tail) and human lactoferrin. After hybrid coating immersion in solution with lactoferrin. the osteoblast-like MG-63 cells exhibited significantly better proliferation and adhesion to the ceramic layer. The hybrid ceramic layer showed the worst adhesion to the substrate compared to only anodized Ti alloy; however, the layer was not brittle and still well-adhered. Formation of multilayer coatings may induce the osseointegration process and further investigations may be developed to make implants for in vivo experiments. This study has some limitations, including analysis of the concentration of adsorbed proteins on the surfaces, stability of the proteins, analysis of the osteoinductive and osteoconductive properties and evaluation the biocompatibility of the coated titanium alloy in in vivo experiments.

## Figures and Tables

**Table 1:** Surface treatment and sample labels of the investigated samples**.**

Sample Label	Anodization	Sol-Gel	Immersion in Collagen Type I Solution	Immersion in Lactoferrin Solution
TAN	-			
TAN-A	√	-		
TAN-SG	√	√	-	
TAN-SG-C	√	√	√	-
TAN-SG-L	√	√	√	√
